# M2 Macrophage-Based Prognostic Nomogram for Gastric Cancer After Surgical Resection

**DOI:** 10.3389/fonc.2021.690037

**Published:** 2021-08-12

**Authors:** Jianwen Hu, Yongchen Ma, Ju Ma, Yanpeng Yang, Yingze Ning, Jing Zhu, Pengyuan Wang, Guowei Chen, Yucun Liu

**Affiliations:** ^1^Department of General Surgery, Peking University First Hospital, Beijing, China; ^2^Endoscopy Center, Peking University First Hospital, Beijing, China

**Keywords:** gastric cancer, macrophage, nomogram, prognosis, M2

## Abstract

A good prediction model is useful to accurately predict patient prognosis. Tumor–node–metastasis (TNM) staging often cannot accurately predict prognosis when used alone. Some researchers have shown that the infiltration of M2 macrophages in many tumors indicates poor prognosis. This approach has the potential to predict prognosis more accurately when used in combination with TNM staging, but there is less research in gastric cancer. A multivariate analysis demonstrated that CD163 expression, TNM staging, age, and gender were independent risk factors for overall survival. Thus, these parameters were assessed to develop the nomogram in the training data set, which was tested in the validation and whole data sets. The model showed a high degree of discrimination, calibration, and good clinical benefit in the training, validation, and whole data sets. In conclusion, we combined CD163 expression in macrophages, TNM staging, age, and gender to develop a nomogram to predict 3- and 5-year overall survivals after curative resection for gastric cancer. This model has the potential to provide further diagnostic and prognostic value for patients with gastric cancer.

## Introduction

More than one million new cases and 783,000 deaths from gastric cancer occurred in 2018. Gastric cancer is the third leading cause of cancer death (1). The degree of tumor invasiveness, number of positive lymph nodes, and distant metastasis are important factors influencing the prognosis of patients with gastric cancer. As the concept of precision medicine gains popularity, we need to more comprehensively explore the factors that influence and predict the prognosis of patients with gastric cancer.

Emerging studies have highlighted that macrophages are important regulators of therapeutic responses and tumor progression, including proliferation, invasion, and apoptosis (1–5). Macrophages are usually of two different subtypes: M1 and M2. M1 macrophages secrete pro-inflammatory cytokines that suppress pathogenic infection and fight against malignancy. M1 macrophage surface markers include the human leukocyte antigen DR isotype (HLA-DR), nitric oxide synthase, and so on (2, 5–8). M2 macrophages secrete various anti-inflammatory molecules that can inhibit the immune system and promote tumor angiogenesis, proliferation, invasion, and metastasis, prevent apoptosis and can be induced by treatment with IL-4 or IL-13 (5, 9, 10). M2 macrophage surface markers include CD163, stabilin-1, CD206, and so on (4, 9, 11).

Many studies have shown that HLA-DR can be used as a marker for M1 macrophages (12–16), CD163 for M2 macrophage (17–19), and CD68 for pan-macrophages (17, 20–22). CD163 is mainly used as an M2 markers in clinical research. CD163+ tumor-associated macrophages infiltration positively correlates with angiogenesis, lymph angiogenesis, reduced overall survival rate, and recurrence-free survival in gastric cancer (23–26).

Although some studies have explored the correlation between intertumoral macrophages and prognosis (2–5), the results were heterogeneous. Based on the characteristics of the immune state of polarized macrophages, immune status can provide a stratification method to more accurately predict prognosis. Combining the microenvironmental indicator macrophage status with a tumor-cell centered stratification system provides a more precise prognostic prediction.

An alignment diagram, also known as a nomogram, is based on multivariate regression analysis. Nomograms integrate multiple forecast indicators and use line segments with scales according to a certain parameter drawn on the same plane to express the relationship among variables in a prediction model. Currently, nomograms are widely applied in major types of cancer (6–11). In addition, nomograms are preferred compared with traditional staging systems for many types of cancer. It is because of the intuitive and easy-to-understand features of nomograms that they have gradually received more and more attention in medical research and clinical practice. Thus, the value of nomograms to predict tumor prognosis is promising (11, 27).

This study aimed to create a postoperative survival model for patients with cancer to predict survival time after resection and to simultaneously explore the prognostic value of macrophage markers using gastric cancer samples. We used a nomogram to transform the complex regression equation into a visual graph, making the results of the prediction model more readable and convenient for evaluation.

## Materials and Methods

### Clinical Specimens and Study Design

A total of 112 patients with primary gastric cancer who underwent standard gastrectomy with lymph node dissection from 2008 to 2013 at Peking University First Hospital were enrolled. All patients underwent regular follow up for 7 to 10 years. Patients were observed until October 2019. Patients undergoing neoadjuvant treatment before surgery were excluded. The pathological diagnostic results were confirmed by two independent gastroenterology pathology doctors, and the gastric cancer classifications were made based on the 8th TNM staging classification for gastric cancer. The clinicopathological characteristics of the 112 patients are shown in **Supplementary Table 1**. The study was approved by the Peking University First Hospital Biomedical Research Ethics Committee. All patients enrolled in the study provided written informed consent. Clinical information, expression of macrophage markers, and follow-up information from 2008 to 2011 (total of 67 patients) were divided into the training set, and stepwise regression was used to screen the most streamlined clinically meaningful indicators to build a prognostic survival forecasting model. At the same time, clinical information of patients from 2011 to 2013 (total of 45 patients) was used as the validation set. The value of postoperative survival prediction in patients with gastric cancer was evaluated in the training, validation, and overall data sets from the viewpoints of discrimination (concordance index), calibration, and social benefits (net benefit). The study design flow chart is shown in **Supplementary Figure 1**.

### Immunohistochemistry

We performed immunohistochemical staining for macrophage markers on all tumor tissues obtained from patients. Murine anti-human CD68 (1:200; Santa Cruz Biotechnology, CA, USA), sc-17832, rabbit anti-human HLA-DR (1:1000; ab92511, Abcam, Cambridge, England) and rabbit anti-human CD163 (1:1000; ab182422, Abcam, Cambridge, England) antibodies were used to confirm the position of macrophages with respect to cancer tissues. Horseradish peroxidase-conjugated goat anti-rabbit and anti-murine IgG (ZB-2305, Zsgb Bio, Beijing, China) were used as secondary antibodies. Macrophage morphology and spatial distribution were assessed by two independent pathologists blinded to patients’ information. A computerized imaging system, including an Olympus DP71 camera and Olympus BX51 microscope, was used to evaluate staining. To ensure homogeneity and representativeness, we scanned immunohistochemistry sections at a high magnification (×400) and captured five independent microscopic fields. The same settings were applied for each image capture. ImagePro Plus version 10.0 (Media Cybernetics, Bethesda, MD, USA) was used to measure the density of macrophage makers. The median relative density was used as the cut-off value for high and low expression. We measured the integrated optical density of all markers that stained positive in each image. We then calculated the ratio of integrated optical density to the total area of each image to obtain the relative density. Results were obtained by calculating the average density of five microscopic fields.

### Statistical Analysis

Stepwise regression algorithms were used to filter variables and build several Cox regression models using Stata for Windows (Stata Corp, TX, USA). We performed a univariate analysis to identify potential risk factors. A P value ≤0.2 was used to select variables for the model. A multivariate analysis was performed to confirm the best-fit model after potential risk factor selection. We used a Cox regression model to select variables to construct a prediction model. We identified the Akaike information criterion (AIC) of these models, selected the variable with the lowest AIC value for the prediction model, and considered the practical clinical significance of the included variables. A nomogram was constructed based on a multivariate Cox regression analysis for further analysis. We used the likelihood ratio test to apply forward stepwise selection with AIC as the stopping rule (28). Discrimination was evaluated using the concordance index, and the calibration curve was evaluated using the unreliability U test (29). The rms package was used for the nomogram and calibration curve in RStudio version 3.5.1 (R Foundation for Statistical Computing, Oakland, New Zealand). To evaluate the clinical benefit of the diagnostic model, a decision curve analysis plot was developed. The net benefit of making the decision was measured using the following formula:

$$\text{Net benefit}=\frac{\text{True positives}}{\text{n}}-\frac{\text{Pt}}{1-\text{Pt}}\times \frac{\text{False positives}}{\text{n}}$$

where n is the total number of patients and Pt is a given threshold probability. Decision Curve Analysis quantified the net benefits at different threshold probabilities in the validation set to determine the clinical usefulness of the nomogram (30, 31). All statistical analyses noted above were two-sided, and P ≤ 0.05 was considered statistically significant. Stata 15.0 for Windows was applied to carry out the calculation.

The correlation between macrophage marker expression and clinicopathology was evaluated using the χ^2^ test for categorical data, the Mann–Whitney U test for continuous and ordinally distributed data, and the Kruskal–Wallis test and Fisher’s exact test for ranked ordinal data. We performed a Kaplan–Meier analysis to calculate the survival duration and used the Breslow test (generalized Wilcoxon test) to analyze the significance between groups. The log-rank test was applied to compare survival between subgroups. A one-sample Kolmogorov-Smirnov test was used to test for normality. A multivariate analysis was performed using the stepwise Cox regression model. Variables with a P value ≤0.2 in the univariate analysis were included in the multivariate regression models. Analysis of Cox regression was employed to compute multivariate hazard ratios and 95% confidence intervals. The results of these tests were analyzed using SPSS version 24.0 (IBM Corp, Armonk, NY, USA). All statistical tests were two-tailed.

## Results

### Macrophage Marker Expression

**Figure 1:**
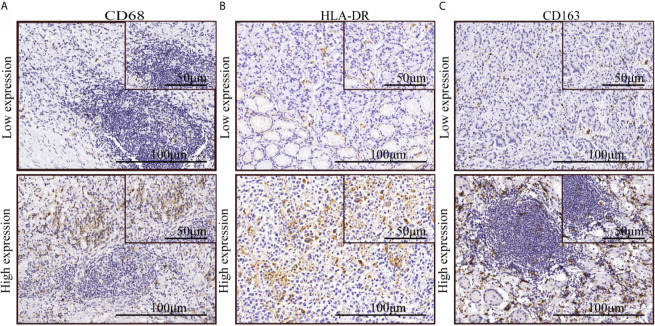
The expression of macrophage markers in gastric cancer. **(A–C)** Immunohistochemical staining indicated low and high expression of CD68, HLA-DR, and CD163.

We applied immunohistochemical methods to observe macrophage subtypes, distribution, and density in tumor areas in samples from patients with gastric cancer. The results showed that CD68 (macrophage marker), HLA-DR (M1 macrophage marker), and CD163 (M2 macrophage marker) were expressed in the cell membrane and cytoplasm. Macrophages were mostly distributed around tumor cell clusters in dense tumor tissues and were scattered in loose tumor tissue. CD68 and CD163 were occasionally positive in tumor tissues from some patients. The degree of expression of different macrophage markers is shown in **Figures 1A–C**. The density of macrophage infiltration in the tumor nests was varied.

### Risk Factors for Overall Survival

A univariate survival analysis indicated that the following clinical factors (*P* = 0.026) were significantly correlated with a reduction in overall survival: T stage (*P* < 0.001), N stage (*P* < 0.001), M stage (*P* < 0.001), nerve invasion (*P* = 0.014), cancer embolus (*P* = 0.039), and CD163 positive macrophage density (*P* = 0.008). A multivariate analysis demonstrated that age (*P* = 0.033), T stage (*P* < 0.001), M stage (*P* < 0.001), and CD163 positive macrophage density (*P* = 0.043) were independent risk factors for overall survival (**Supplementary Table 2**). Therefore, infiltration of M2 macrophages in gastric cancer has the potential to predict prognosis.

### Clinicopathological Characteristics of the Training and Validation Sets

**Table 1** shows the clinical features and expression of macrophage markers, which were collected and included in stepwise screening. To facilitate a comprehensive understanding of the data, TNM stages are also shown in the table, but this variable was not included in the stepwise method to avoid repetition. Sixty-seven samples were included in the training set, which were collected from patients with an average age of 63.7 ± 12.1 years and a median age of 64.0 years. Forty-five samples were obtained from patients in the validation set with an average age of 61.7 ± 12.9 years and a median age of 65.0 years. The median survival times of patients in the training and validation sets were 73.0 and 49.0 months, respectively, and the 5-year survival rates were 52.2% and 46.7%, respectively.

**Table 1:** Clinicopathological characteristics of the training and validation sets.

Factors	**Training**		**Validation**	
****	No.	%	No.	%
**Mean age (years)**				
**Age (mean ± standard deviation, years)**	63.7 ± 12.1		61.7 ± 12.9	
**Gender**				
**Female**	17	25.4	11	24.4
**Male**	50	74.6	34	75.6
**Surgery type**				
**Total gastrectomy**	34	73.9	34	51.5
**Subtotal gastrectomy**	12	26.1	32	48.5
**Tumor Size (cm)**				
**<5**	30	44.8	10	22.2
**≧5**	37	55.2	35	77.8
**Pathological type**				
**Adenocarcinoma**	41	61.2	29	64.4
**Mucinous carcinoma**	4	6.0	2	4.4
**Silver ring cell carcinoma**	22	32.8	14	31.1
**Bormann classification**				
**I**	4	6.0	2	4.4
**II**	30	44.8	9	20.0
**III**	28	41.8	29	64.4
**IV**	5	7.5	5	11.1
**Grade**				
**Poorly**	51	76.1	37	82.2
**Moderate**	13	19.4	8	17.8
**Good**	3	4.5	0	0
**T Stage**				
**T1**	5	7.5	0	0
**T2**	5	7.5	0	0
**T3**	32	47.8	18	40
**T4**	25	37.3	27	60
**N Stage**				
**N0**	19	28.4	5	11.1
**N1**	8	11.9	8	17.8
**N2**	14	20.9	13	28.9
**N3**	26	38.8	19	42.4
**M Stage**				
**No**	60	89.6	41	91.1
**Yes**	7	10.4	4	8.9
**TNM classification**				
**I**	5	7.5	0	0
**II**	5	7.5	0	0
**III**	32	47.7	18	40.0
**IV**	25	37.3	27	60.0
**Nerve invasion**				
**No**	35	52.2	12	26.7
**Yes**	32	47.8	33	73.3
**Cancer embolus**				
**No**	51	76.1	27	60
**Yes**	16	23.9	18	40
**CD68 expression**				
**Low**	34	50.7	22	48.9
**High**	33	49.3	23	51.1
**HLA-DR expression**				
**Low**	28	41.8	28	62.2
**High**	39	58.2	17	37.8
**CD163 expression**				
**Low**	34	50.7	22	48.9
**High**	33	49.3	23	51.1

Multivariate analyses with procedure forward, backward, and stepwise were performed to establish the best-fit model in the training set. After calculating and comparing the AIC values of these models, we selected the model with the lowest AIC value (AIC = 238.1). As shown in **Table 2**, the prediction model included five variables (age, T stage, N stage, M stage, and CD163 expression level) with practical clinical significance.

**Table 2:** Predictors of overall survival in patients with gastric cancer.

Variable	Coef.	Stderr	Z	P	95%CI	
**Gender**	1.354	0.555	2.44	0.015	0.266	2.442
**Age**	0.033	0.018	1.84	0.066	−0.002	0.698525
**T stage**	1.219	0.335	3.64	0.000	0.564	1.875
**N stage**	0.251	0.164	1.53	0.127	−0.710	0.573
**M stage**	1.849	0.517	3.58	0.000	0.836	2.862
**CD163 Expression**	0.552	0.398	1.39	0.165	−0.228	1.332

### Prognostic Nomogram for Overall Survival in Patients With Gastric Cancer

**Figure 2:**
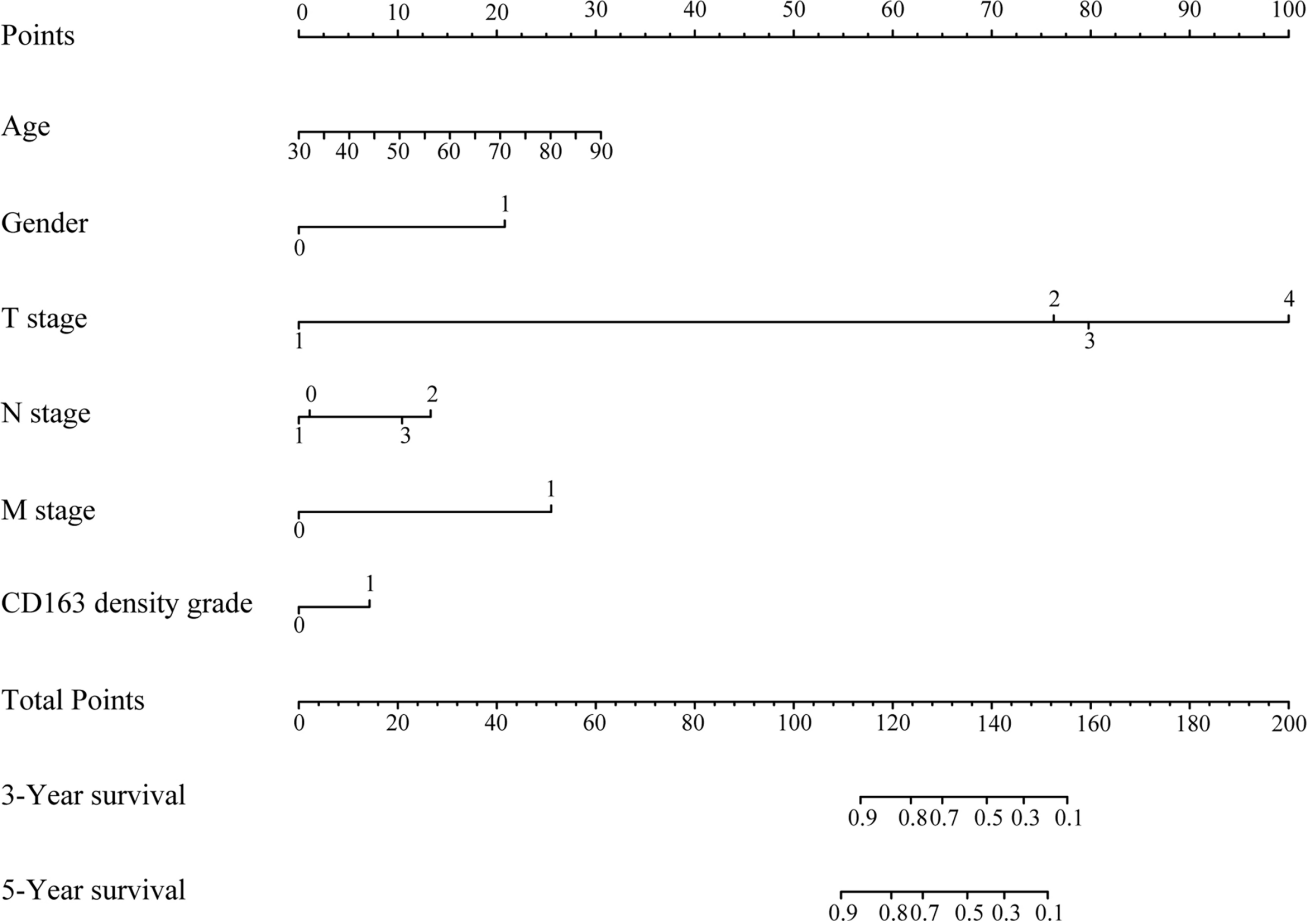
Prognostic nomogram for overall survival of patients with gastric cancer. The total score was the sum of the individual scores for every variable, and the length of the line segment reflected how much the factor contributed to the outcome events. Three- and 5-year survival in the nomogram indicated the survival probabilities.

The variables included in the prediction model were age, gender, TNM stage, and density of CD163 expression (**Figure 2**). The basic principle of the nomogram is to construct multi-factor Cox regression according to the contribution of each influencing factor in the model to the outcome variables (3- and 5-year survival after gastric resection). The scores of each influencing factor were then summed to obtain the total score, and the individual outcomes were calculated based on the functional conversion relationship between the total score and the probability (the predicted value) of 3- and 5-year survival events.

### Performance of the Model in the Training, Validation, and Overall Data Sets Concordance Index and Calibration Plot

**Figure 3:**
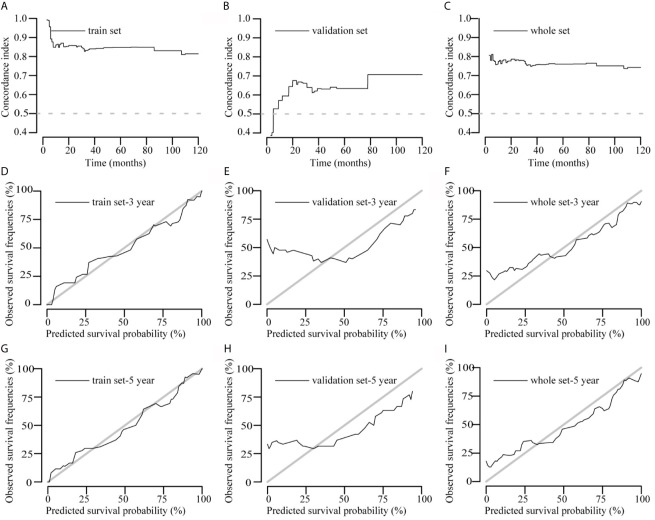
Concordance index (c-index) and calibration curves of the model. **(A–C)** C-index plots of the model in the training, validation, and overall data sets, respectively. **(D–I)** Calibration curves of the model to predict 3- and 5-year survival rates in the training, validation, and overall data sets, respectively. Actual survival is plotted on the y axis, and the x axis represents the predictive probability of the model.

Concordance index (c-index) plots are shown in **Figures 3A–C**. We applied the constructed model to the training set, validation set, and the overall data set to judge the discrimination of this model to predict survival time during the follow-up period (0–120 months). The model showed a high degree of discrimination in all three data sets (c-index, >0.6 from 12 to 120 months).

The calibration curve graphically represents the correlation between the predicted probabilities and the observed outcome frequencies. The degree of conformity between the calibration curve and the standard curve represents the degree of calibration. Calibration of 3- and 5-year survival in the three data sets is shown in **Figures 3D–I**. The 5-year survival calibration curves of this model were a very good fit with the standard curve in all three data sets, and 3-year survival calibration curves were only good fit with the standard curve in training and overall data sets.

### Decision Curve Analysis Plots

**Figure 4:**
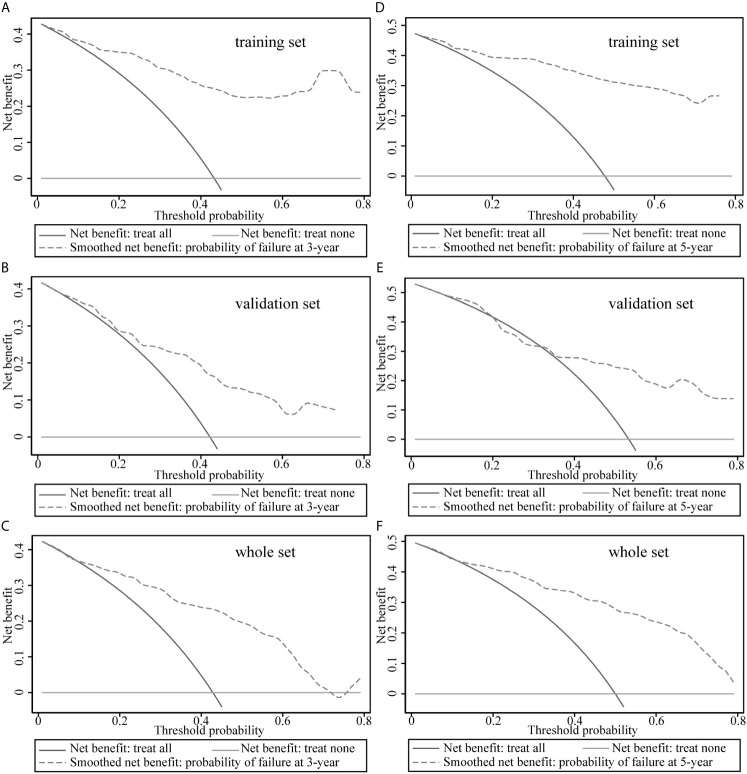
Decision curve analysis of the model. **(A–C)** Decision curve analysis to predict 3-year survival rates in the training, validation, and overall data sets. **(D–F)** Decision curve analysis plots to predict 5-year survival rates in the training, validation, and overall data sets.

The decision curves for the model are illustrated in **Figure 4**. The clinical impact of the prediction model to identify individuals for survival prediction was observed at thresholds of ≥18% for 3-year survival and ≥30% for 5-year survival. The model was useful between threshold probabilities of 10% and 75% for 3-year survival and 35% and 80% for 5-year survival.

### Relationship Between Macrophage Polarization and Clinicopathological Features

**Figure 5:**
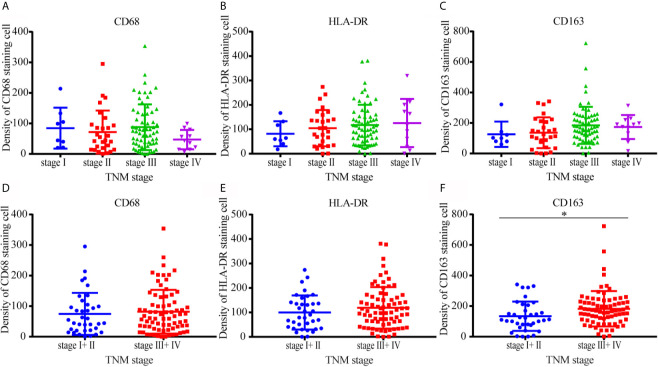
Distribution of CD68, HLA-DR, and CD163 positive macrophages in different TNM stages. **(A–C)** There was no significant difference in the density of CD68, HLA-DR, and CD163 among samples of different TNM stages I, II, III, and IV. **(D, E)** The distribution trends of CD68 and HLA-DR expression between stage I–II and III–IV were same. **(F)** The density of CD163 in M2 macrophages was higher in TNM stage III–IV compared with TNM stage I–II.

The correlation between CD68, the expression of other molecules, and clinicopathological features was shown in **Supplementary Table 1**. CD163 staining was correlated with the invasion of lymph nodes (*P* = 0.002), TNM stage (*P* = 0.017), and tumor size (*P* = 0.049). There was no significant difference in the density of CD68, HLA-DR, and CD163 among samples of different TNM stages I, II, III, IV (**Figures 5A–C**). Same distribution trends of CD68 and HLA-DR expression between stage I–II and III–IV were shown in **Figures 5D, E**. The density of CD163 in M2 macrophages was higher in TNM stage III–IV compared with TNM stage I–II (**Figure 5F**). There was no significant difference in the density distribution of other types of macrophage between different TNM stages in this study.

### Prognostic Value of Macrophage Polarization

**Figure 6:**
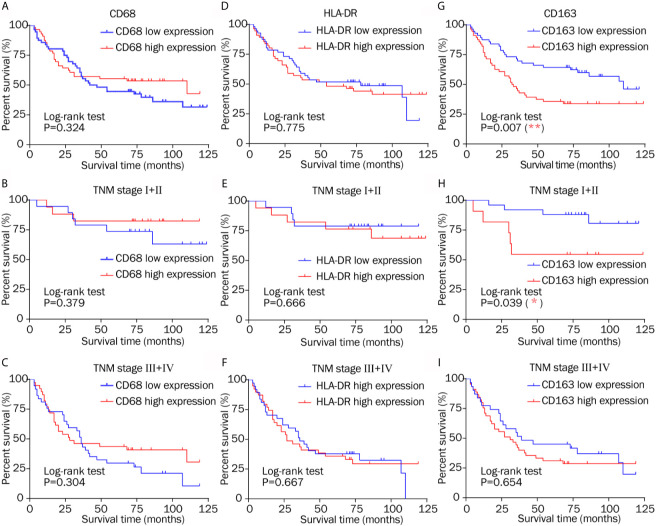
Kaplan–Meier survival curves of low and high expression of CD68, HLA-DR, and CD163 in different TNM stages. **(A–C)** CD68 staining had no obvious correlation with overall survival in different TNM stage. **(D–F)** Similarly, HLA-DR showed no evident relationship with overall survival in patients in different stages. **(G)** High CD163 expression was related to reduced survival. **(H)** High CD163 expression indicated a shorter survival time after resection in TNM stage I–II. **(I)** There was no different overall survival between low and high CD163 expression in TNM stage III–IV.

A Kaplan–Meier analysis was performed on the overall data set. High CD163 expression was related to reduced survival (**Figure 6G**; *P* = 0.007), whereas CD68 and HLA-DR staining had no obvious correlation with overall survival (**Figures 6A, D**; *P* = 0.324 and *P* = 0.775, respectively).

High CD163 expression indicated a shorter survival time after resection in TNM stage I–II (**Figure 6H**; *P* = 0.039), which is consistent with the conclusions of others (2, 23, 32). CD68 showed no significant relationship with overall survival in patients classified as TNM stage I–II or TNM stage III–IV (**Figures 6B, C**; *P* = 0.379, and *P* = 0.304, respectively). Zhang’s research came to the same conclusion 2. Similarly, HLA-DR showed no evident relationship with overall survival in patients in the early and advanced stages (**Figures 6E, F**; *P* = 0.666 and *P* = 0.667, respectively). The results do not illustrate that total macrophage and M1 macrophage infiltration had prognostic significance, which is different from other studies (2, 25). These results only suggest that M2 makers, but not M1 or macrophage markers, offer prognostic information in different TNM stages.

## Discussion

The type and function of immune cells play an important role in tumor development (33, 34). Inflammation in the tumor microenvironment is an important manifestation of malignant tumors, and macrophages are the main inflammatory cells in the tumor microenvironment. Monocytes are derived from CD34 primary myeloid progenitor cells in the bone marrow, are distributed in the blood circulation, and enter tissues to differentiate into different macrophage subtypes (35–37). M2 macrophages indirectly promote tumor cell growth by secreting interleukin-10 and transforming growth factor-β while inhibiting immune response cells in the microenvironment. However, the impact of macrophage infiltration in the tumor microenvironment is still controversial. Some researchers have shown that massive infiltration of tumor tissues causes excessive macrophages to participate in the inflammatory response, providing an environment that supports tumor growth and metastasis (38–41).

In this study, we explored the prognostic significance of M1/M2 macrophage markers in patients with gastric cancer and found that an increase in M2 macrophages was the main reason for malignant development of tumors, whereas the overall density of macrophages and the density of M1 macrophages showed no effect on the prognosis of patients with gastric cancer. Univariate and multivariate Cox regression analyses demonstrated that age, TNM stage, and CD163 were independent risk factors for overall survival. Therefore, infiltration of M2 macrophages in gastric cancer has the potential to predict prognosis, which is consistent with the results reported by other researchers in other tumor types.

The TNM staging system is the most common method for predicting patient prognosis. The incidence of gastric cancer and the mortality associated with gastric cancer are higher in males than in females (1). Younger patients are generally recognized to have longer overall survival compared with older patients with gastric cancer (42). At the same time, a Korean study showed that in patients with stage I–II gastric cancer, the <50-year age group was associated with a significantly lower proportion of cancer-specific mortality compared with the 70–79-year age group (43). These studies suggest that if TNM staging is combined with M2 marker expression, age, and gender, this would greatly improve the prognostic ability of postoperative survival.

In the present study, we established a nomogram to evaluate the survival probability of patients after gastric cancer resection. We incorporated the recognized TNM staging system and the degree of M2 infiltration, age, and gender into this model, increasing the predictive accuracy of the model. Considerable differences in survival were observed even in patients with the same stage of gastric cancer. When the constructed model was applied to the validation and overall data sets, a high degree of discrimination and calibration were verified. And it was equipped with a high clinical benefit rate. The postoperative survival prediction model has good extrapolation potential.

The correlation analysis between macrophage polarization status and clinicopathological stage in gastric cancer showed that the density of CD163-positive macrophages was higher in patients with TNM III–IV compared with patients with TNM stage I–II. The density differences among other types of macrophage towards TNM staging did not reach significance in the present study. The prognostic survival analysis demonstrated that high expression of CD163 was related to reduced overall survival after resection in patients with early-stage tumors, which is consistent with the conclusions of others (17–19). These results fully explain why infiltration of M2 macrophages can predict prognosis and survival time when combined with TNM staging.

Zhang et al.’s study shows that CD68+ tumor-associated macrophages in gastric cancer have no significant association with overall survival and Kim et al.’s study also illustrates CD68+ tumor-associated macrophages have no prognostic impact on disease-free survival in MSI-H advanced gastric cancer (32), which are the same as our conclusion. Zhang et al. used CD206 as a marker for M2, and its high expression predicted a worse prognostic survival and was included in the nomogram model, whereas we used CD163 as a marker for M2 to conduct research. Both CD206 and CD163 are more recognized markers for M2, and the conclusion we have reached that is similar to Zhang’s conclusion. However, Zhang’s results also show that the high expression of M1 marker CD11 predicts better survival time. Our results did not find that the high expression of M1 marker HLA-DR had prognostic significance, and it was not included in the nomogram model. It may be caused by our small sample size. However, some people’s research also has the opposite conclusion. Wang et al.’s study shows that the intra-tumoral infiltration of CD68+ tumor-associated macrophages is an independent good prognostic factor in gastric cancer (44). Similarly, Wu et al.’s study demonstrates that CD68+ tumor-associated macrophages promote angiogenesis and lymph angiogenesis of gastric cancer (26). Additionally, Ishigami et al. argues that patients with a high count of CD68+ tumor-associated macrophages have poorer surgical outcomes than those with a low count (45). The prognostic significance of CD68-positive macrophages is still controversial in gastric cancer, and a larger sample size is needed to clarify its prognostic significance.

The results of the present study might provide evidence for clinicians to stratify patients with different prognoses. Due to the limitations of single-center retrospective studies, more multi-center studies are needed to further validate the effects of macrophage infiltration into the tumor microenvironment and to determine the efficacy of the M2 macrophage-based nomogram on the prognosis of patients with gastric cancer.

## Conclusion

In conclusion, we combined CD163 expression with TNM staging, age, and gender to construct a nomogram that has the potential to be used as a useful prognostic tool for patients with gastric cancer. An increase in M2 macrophages predicts a shorter survival time in patients after surgery for gastric cancer. This study provides a theoretical basis and highlights potential targets for immunotherapy in patients with gastric cancer.

## Data Availability Statement

The original contributions presented in the study are included in the article/**Supplementary Material**. Further inquiries can be directed to the corresponding authors.

## Ethics Statement

The study was approved by the Peking University First Hospital Biomedical Research Ethics Committee (No.2017-37). All patients connected with this study signed an informed consent agreement. The patients/participants provided their written informed consent to participate in this study.

## Author Contributions 

JH and YM participated in the study design, carried out the immunocytochemical staining and analysis, and completed the manuscript. YY and YN performed clinical information collection from patients. JZ analyzed patient data and PW performed the statistical analysis. YL and GC were major contributors to the design of this study and revised the manuscript. All authors contributed to the article and approved the submitted version.

## Funding

The National Natural Science Foundation of China (No. 81902384 and No. 81770522) and National Science and Technology Major Project of China (2018ZX10723204) supported the completion of this project.

## Conflict of Interest

The authors declare that the research was conducted in the absence of any commercial or financial relationships that could be construed as a potential conflict of interest.

## Publisher’s Note

All claims expressed in this article are solely those of the authors and do not necessarily represent those of their affiliated organizations, or those of the publisher, the editors and the reviewers. Any product that may be evaluated in this article, or claim that may be made by its manufacturer, is not guaranteed or endorsed by the publisher.

## Supplementary Material

Supplementary Figure 1 Design flowchart of the study.

## Abbreviations

M1, M1-type tumor-associated macrophage; M2, M2-type tumor-associated macrophage; HR, Hazard ratio.
