# Supplementary figures and images for: M2 Macrophage-Based Prognostic Nomogram for Gastric Cancer After Surgical Resection
Source: Front Oncol. 2021 Aug 12;11:690037. doi: 10.3389/fonc.2021.690037 (PMC8397443; doi:10.3389/fonc.2021.690037)

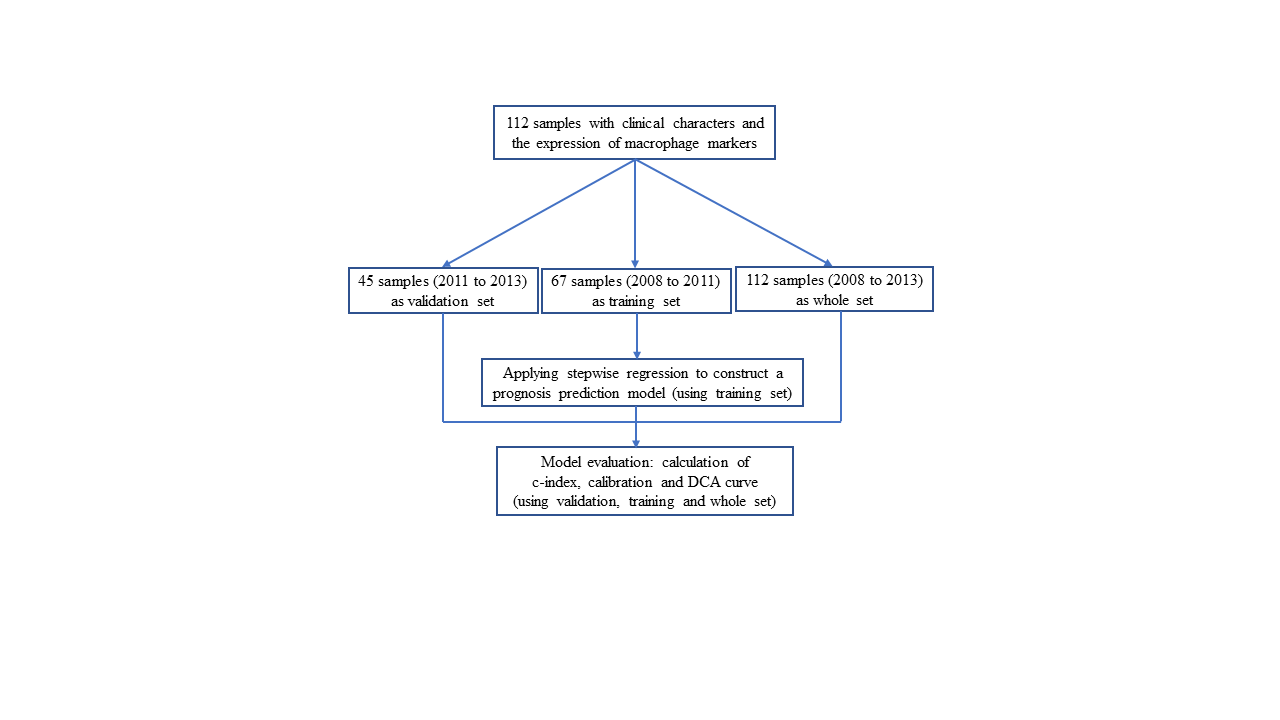

Supplement: Supplementary Figure 1 — Design flowchart of the study. [file Image_1.tif]
