# Supplementary material for: M2 Macrophage-Based Prognostic Nomogram for Gastric Cancer After Surgical Resection
Source: Front Oncol. 2021 Aug 12;11:690037. doi: 10.3389/fonc.2021.690037 (PMC8397443; doi:10.3389/fonc.2021.690037)
Supplement: Supplementary file 2 [file Table_1.docx]

**Supplementary table 1**: Correlation between clinical data and macrophage marker expression.

|  | Patients | CD68 expression | | | HLA-DR expression | | | CD163 expression | | |
| --- | --- | --- | --- | --- | --- | --- | --- | --- | --- | --- |
| Factors | No. (%) | Low (n=45) | High (n = 45) | *P* | Low (n = 45) | High (n= 45) | *P* | Low(n=45) | High (n= 45) | *P* |
| Age (mean ± standard deviation） | 62.9±12.4 |  |  |  |  |  |  |  |  |  |
| Gender |  |  |  |  |  |  |  |  |  |  |
| Female | 28 (25.0) | 13(46.4) | 15(53.6) | 0.690 | 14(50.0) | 14(50.0) | 1.00 | 15(53.6) | 13(46.4) | 0.663 |
| Male | 84 (75.0) | 43(51.2) | 41(48.8) |  | 42(50.0) | 42(50.0) |  | 41(48.8) | 43(51.2) |  |
| Surgery type |  |  |  |  |  |  |  |  |  |  |
| Total gastrectomy | 68(60.7) | 36(52.9) | 38(47.1) | 0.562 | 33(48.5) | 35(51.5) | 0.847 | 31(45.5) | 37(54.5) | 0.246 |
| Subtotal gastrectomy | 44(39.3) | 20(45.4) | 24(54.5) |  | 23(52.2) | 21(47.8) |  | 25(56.8) | 19(43.2) |  |
| Age |  |  |  |  |  |  |  |  |  |  |
| <60 years | 38(33.9) | 18(47.4) | 20(52.6) | 0.663 | 19(50.0) | 19(50.0) | 1.00 | 23(60.5) | 15(39.5) | 0.110 |
| ≥60 years | 74(66.1) | 38(51.4) | 36(48.6) |  | 37(50.0) | 37(50.0) |  | 33(44.6) | 41(55.4) |  |
| Tumor size (cm) |  |  |  |  |  |  |  |  |  |  |
| <5 | 40(35.7) | 20(50) | 20(50) | 1.000 | 23(57.5) | 17(42.5) | .237 | 25(62.5) | 15(37.5) | **0.049** |
| ≧5 | 72(64.3) | 36(50) | 36(50) |  | 33(45.8) | 39(54.2) |  | 31(43.1) | 41(56.9) |  |
| Pathological type |  |  |  |  |  |  |  |  |  |  |
| Adenocarcinoma | 70(62.5) | 38(54.3) | 32(45.7) | 0.450 | 37(52.9) | 33(47.1) | 0.627 | 34(48.6) | 36(51.4) | 0.153 |
| - Mucinous carcinoma | 6(5.4) | 2(33.3) | 4(66.7) |  | 2(33.3) | 4(66.7) |  | 1(16.7) | 5(83.3) |  |
| - Silver ring cell carcinoma | 36(32.1) | 16(44.4) | 20(55.6) |  | 17(47.2) | 19(52.8) |  | 21(58.3) | 15(41.7) |  |
| Bormann classification |  |  |  |  |  |  |  |  |  |  |
| Ⅰ | 6(5.4) | 3(50) | 3(50) | 0.900 | 5(83.3) | 1(16.7) | 0.214 | 3(50.0) | 3(50.0) | 0.375 |
| Ⅱ | 39(34.8) | 20(51.3) | 19(48.7) |  | 22(56.4) | 17(43.6) |  | 24(61.5) | 15(38.5) |  |
| Ⅲ | 57(50.9) | 27(47.4) | 30(52.6) |  | 25(43.9) | 32(56.1) |  | 25(43.9) | 32(56.1) |  |
| Ⅳ | 10(8.9) | 6(60.0) | 4(40.0) |  | 4(40.0) | 6(60.0) |  | 4(40.0) | 6(60.0) |  |
| Grade |  |  |  |  |  |  |  |  |  |  |
| Poorly | 88(78.5) | 43(48.9) | 45(51.1) | 0.719 | 47(53.4) | 41(46.6) | 0.353 | 43(48.9) | 45(51.1) | 0.719 |
| Moderate | 21(18.8) | 12(57.1) | 9(42.9) |  | 7(33.3) | 14(66.7) |  | 12(57.1) | 9(42.9) |  |
| Good | 3(2.7) | 1(33.3) | 2(66.7) |  | 2(66.7) | 1(33.3) |  | 1(33.3) | 2(66.7) |  |
| T Stage |  |  |  |  |  |  |  |  |  |  |
| T1 | 5(4.5) | 3(60.0) | 2(40.0) | 0.389 | 3(60.0) | 2(40.0) | 0.940 | 5(100.0) | 0(0.00) | 0.111 |
| T2 | 5(4.5) | 1(20.0) | 4(80.0) |  | 2(40.0) | 3(60.0) |  | 3(60.0) | 2(40.0) |  |
| T3 | 50(44.6) | 23(46.0) | 27(54.0) |  | 25(50) | 25(50) |  | 24(48.0) | 26(52.0) |  |
| T4 | 52(46.4) | 29(55.8) | 23(44.2) |  | 26(50) | 26(50) |  | 24(46.2) | 28(53.8) |  |
| N Stage |  |  |  |  |  |  |  |  |  |  |
| N0 | 24(21.4) | 15(62.5) | 9(37.5) | 0.591 | 11(45.8) | 13(54.2) | 0.705 | 17(70.8) | 7(29.2) | **0.02** |
| N1 | 16(14.3) | 7(43.8) | 9(56.3) |  | 10(62.5) | 6(37.5) |  | 0(56.3) | 7(43.8) |  |
| N2 | 27(24.1) | 13(48.1) | 14(51.9) |  | 14(51.9) | 13(48.1) |  | 10(37.0) | 17(63.0) |  |
| N3 | 45(40.2) | 21(46.7) | 24(53.3) |  | 21(46.7) | 24(53.3) |  | 20(44.4) | 25(55.6) |  |
| M Stage |  |  |  |  |  |  |  |  |  |  |
| No | 101(90.2) | 48(47.5) | 53(52.5) | 0.112 | 51(50.5) | 50(49.5) | 0.751 | 53(52.5) | 48(47.5) | 0.112 |
| Yes | 11(9.8) | 8(72.7) | 3(27.3) |  | 5(45.5) | 6(54.5) |  | 3(27.3) | 8(72.7) |  |
| TNM classification |  |  |  |  |  |  |  |  |  |  |
| Ⅰ | 8(7.1) | 4(50.0) | 4(50.0) | 0.375 | 5(62.5) | 3(37.5) | 0.933 | 7(87.5) | 1(12.5) | **0.017** |
| Ⅱ | 28(25) | 15(53.6) | 13(46.4) |  | 14(50.0) | 14(50.0) |  | 18(64.3) | 10(35.7) |  |
| Ⅲ | 66(58.9) | 29(44.6%) | 36(55.4) |  | 32(49.2) | 33(50.8) |  | 28(43.1) | 37(56.9) |  |
| Ⅳ | 10(9.0) | 8(72.7) | 3(27.) |  | 5(45.5) | 6(54.5) |  | 3(27.3) | 8(72.7) |  |
| Nerve invasion |  |  |  |  |  |  |  |  |  |  |
| No | 47(42.0) | 21(44.7) | 26(55.3) | 0.338 | 21(44.7) | 26(55.3) | 0.338 | 23(48.9) | 24(51.1) | 0.848 |
| Yes | 65(58.0) | 35(53.8) | 30(46.2) |  | 35(53.8) | 30(46.2) |  | 33(50.8) | 32(49.2) |  |
| Cancer embolus |  |  |  |  |  |  |  |  |  |  |
| No | 78(69.6) | 38(48.7) | 40(51.3) | 0.681 | 40(51.3) | 38(48.7) | 0.681 | 41(52.6) | 37(47.4) | 0.411 |
| Yes | 34(30.4) | 18(52.9) | 16(47.1) |  | 16(47.1) | 18(52.9) |  | 15(44.1) | 19(55.9) |  |
