# Supplementary material for: M2 Macrophage-Based Prognostic Nomogram for Gastric Cancer After Surgical Resection
Source: Front Oncol. 2021 Aug 12;11:690037. doi: 10.3389/fonc.2021.690037 (PMC8397443; doi:10.3389/fonc.2021.690037)
Supplement: Supplementary file 3 [file Table_2.docx]

**Supplementary table 2:** Univariate and multivariate Cox regression analysis of prognosis in patients with gastric cancer.

|  | [**Univariate analysis**](javascript:;) | | |  | **Multivariate analysis** | | |  |
| --- | --- | --- | --- | --- | --- | --- | --- | --- |
|  |  | **95% CI** | |  |  | **95% CI** | |  |
|  | **Hazard ratio** | **Lower** | **Upper** | ***P*** | **Hazard ratio** | **Lower** | **Upper** | ***P*** |
| **Age** | 1.025 | 1.003 | 1.047 | **0.026** | 1.026 | 1.000 | 1.051 | **0.048** |
| **Gender** |  |  |  |  |  |  |  |  |
| Female | Reference |  |  |  |  |  |  |  |
| Male | 1.682 | 0.894 | 3.165 | 0.107 | 1.163 | 0.562 | 2.406 | 0.684 |
| **Surgery type** |  |  |  |  |  |  |  |  |
| Subtotal gastrectomy | Reference |  |  |  |  |  |  |  |
| Total gastrectomy | 1.830 | 1.065 | 3.145 | 0.029 | 1.735 | 1.015 | 2.967 | 0.559 |
| **Histological type** |  |  |  |  |  |  |  |  |
| Adenocarcinoma | Reference |  |  |  |  |  |  |  |
| Others | 1.07235 | 0.645 | 1.782 | 0.787 |  |  |  |  |
| **Grade** |  |  |  |  |  |  |  |  |
| Good | Reference |  |  | 0.571 |  |  |  |  |
| Moderate | 1.986 | 0.273 | 14.419 | 0.498 |  |  |  |  |
| Poor | 1.495 | 0.193 | 11.606 | 0.701 |  |  |  |  |
| **Bormann classification** |  |  |  |  |  |  |  |  |
| Ⅰ+Ⅱ | Reference |  |  |  |  |  |  |  |
| Ⅲ+Ⅳ | 1.625719 | .959 | 2.757 | 0.071 | 1.364 | 0.445 | 4.184 | 0.587 |
| **Tumor size** |  |  |  |  |  |  |  |  |
| <5cm | Reference |  |  |  |  |  |  |  |
| ≥5cm | 1.630 | 0.940 | 2.826 | .082 | 0.595 | 0.171 | 2.069 | 0.414 |
| **T stage** |  |  |  |  |  |  |  |  |
| T1-3 | Reference |  |  |  |  |  |  |  |
| T4 | 4.516 | 2.630 | 7.753 | 0.000 | 3.289 | 1.786 | 6.054 | **0.000** |
| **N stage** |  |  |  |  |  |  |  |  |
| N0-N2 | Reference |  |  |  |  |  |  |  |
| N3 | 2.514 | 1.518 | 4.163 | 0.000 | 1.638 | .936 | 2.866 | **0.084** |
| **M Stage** |  |  |  |  |  |  |  |  |
| **No** | Reference |  |  |  |  |  |  |  |
| Yes | 4.281 | 2.200 | 8.330 | 0.000 | 2.444 | 1.200 | 4.978 | **0.014** |
| **TNM classification** |  |  |  |  |  |  |  |  |
| 1 | Reference |  |  | 0.000 |  |  |  |  |
| 2 | 2.383 | 0.298 | 19.067 | 0.413 |  |  |  |  |
| 3 | 7.589 | 1.042 | 55.252 | 0.045 |  |  |  |  |
| 4 | 22.712 | 2.908 | 177.369 | 0.003 |  |  |  |  |
| **Nerve invasion** |  |  |  |  |  |  |  |  |
| No | Reference |  |  |  |  |  |  |  |
| Yes | 1.963 | 1.144 | 3.366 | 0.014 | 1.472 | 0.791 | 2.739 | 0.222 |
| **Cancer embolus** |  |  |  |  |  |  |  |  |
| No | Reference |  |  |  |  |  |  |  |
| Yes | 1.731 | 1.029 | 2.912 | 0.039 | 1.058 | 0.597 | 1.876 | 0.846 |
| **CD68 expression** |  |  |  |  |  |  |  |  |
| Low | Reference |  |  |  |  |  |  |  |
| High | 0.778 | 0.470 | 1.286 | 0.328 |  |  |  |  |
| **HLA-DR expression** |  |  |  |  |  |  |  |  |
| Low | Reference |  |  |  |  |  |  |  |
| High | 1.075 | 0.651 | 1.774 | 0.777 |  |  |  |  |
| **CD163 expression** |  |  |  |  |  |  |  |  |
| Low | Reference |  |  |  |  |  |  |  |
| High | 1.996 | 1.196 | 3.333 | 0.008 | 1.735 | 1.015 | 2.967 | **0.044** |
